# When patrons “click” with the library’s literature searches: using data from a novel link generation process to assess patron engagement with literature search results

**DOI:** 10.29173/jchla29914

**Published:** 2026-08-03

**Authors:** Tyler Ostapyk, Nicole Askin, Carla Epp, Jenna G. Tichon

**Affiliations:** 1 Winnipeg Regional Health Authority Virtual Library, University of Manitoba, Winnipeg, MB; 2 University of Manitoba, Winnipeg, MB

## Abstract

**Introduction:**

One of the core services the Winnipeg Regional Health Authority Virtual Library (WRHAVL) provides is literature searching for healthcare staff. Librarians send results to patrons as a reference list with links to the items identified. To improve access to the library’s collection, and to understand patrons’ engagement, the library developed a novel process for generating trackable links. Link and click data were analyzed to assess patron engagement with the literature search service.

**Methods:**

Leveraging the link management tool Short.io and a Visual Basic for Applications macro, the library uses an automated process to generate trackable links for literature search results sent to patrons. Click and link data are automatically collected in Short.io. Data from 19 October 2022 to 31 October 2024 was cleaned and exported into Excel for analysis. Logistic regression analysis was conducted in R.

**Results:**

Over the two-year study period, librarians sent 344 literature searches to patrons using this method. Patrons clicked at least one link in 194, or 56%, of reference lists. The percentage of total links clicked was higher for point of care and quick reference resources. Greater numbers of digital object identifier (DOI) and PubMed identifier (PMID) links included in a literature search predicted a decrease in the odds of someone clicking at least one link. Patrons clicked more than 20 links in only 10, or just 3%, of literature searches.

**Discussion:**

The data provides valuable insights into patron engagement with the WRHAVL’s literature search service. The discovery that 44% of reference lists did not have a single click warrants further investigation.

## Introduction

The Winnipeg Regional Health Authority Virtual Library (WRHAVL) provides services to hospital and healthcare staff in Winnipeg, Manitoba, Canada. One of the core functions of the library is conducting literature searches for healthcare professionals, providing them with a list of references and abstracts on their topic. In most cases, patrons provide no follow-up response after receiving their literature search results. For this reason, in the past the library found it difficult to determine how patrons were engaging with the results provided.

At the same time, the library’s patrons frequently expressed they found it challenging to access full-text articles, including those identified in literature search results. With the aim of addressing both issues, in 2022 the library began exploring options for a tool or process that would:

1. Improve full-text access links and streamline the process for generating these links.
2. Enable the library to assess patron engagement with the literature search results they receive.

### Improving full-text access to literature search results

As described by Westmark et al., most institutions that provide mediated literature searches include link resolver links or buttons in their search results [1]. These are software tools that establish links between individual citations and the corresponding full-text item in a database or the library’s catalogue [2]. However, after examining the link resolver options used by libraries they surveyed, Westmark et al. found that none of them offered a simple, quick method to provide links in literature search results without requiring requestors to create a citation management account or install a citation manager [1]. The WRHAVL first explored the possibility of utilizing RefWorks’ (Clarivate) shared folder feature to send users results containing full-text buttons. However, like Westmark et al., the library discovered that RefWorks requires recipients to log in to personal RefWorks accounts to view abstracts and link-resolver buttons in shared folders [1]. As the library provides services to over 15,000 individuals across several sites, it was not feasible to license RefWorks or to ask patrons to obtain RefWorks accounts. Additionally, RefWorks representatives confirmed in an e-mail [3] and a subsequent meeting [4] that the statistics provided by RefWorks were not granular enough for the library to assess engagement at the level of an individual literature search.

Similar to the approach developed by Westmark et al. [1], the library had been using a Visual Basic for Applications (VBA) macro in Word to generate link resolver links (i.e. OCLC EZproxy links) based on digital object identifiers (DOIs) and PubMed identifiers (PMIDs). However, when the full text was not available through the library’s collection, the link would often point the patron to the publisher’s website where they were unable to access the article and/or were prompted to pay a fee for access. This caused confusion for patrons who then had to navigate back to the library website or catalogue to request the items through the library.

To improve these links, the library explored the use of LibKey (Third Iron) [5]. Using LibKey Link Advanced Linking Routes [6], the library was able to create library-specific links based on DOIs and PMIDs. These links prompt the patron to log in to their library account and then open the full-text PDFs when the resource is available in the collection. When the resource is not available, the links redirect the patron to a record for the item in the library’s discovery service (Ex Libris Primo), where they can request the full text. Altering the macro to use these links resolved the issue concerning the EZproxy links, as the links now directed patrons to the Primo record when items were not available. Although Third Iron does provide some statistics for LibKey, as with RefWorks they are not granular enough to look at engagement at the individual literature search level, so additional options were explored for assessing patron engagement with the results they received.

### Assessing patron engagement with literature search services

There have been several studies evaluating literature search services for health professionals since the early 2000s, but they broadly fall into one of two categories:

1. Analysis of literature search request/ completion data [7-13]
2. Surveys of service users [14-31]

Beyond these methods, two studies have assessed literature search services by comparing results produced for the same question under different contexts: mediated (i.e. assisted by a search specialist) vs. unmediated searches (i.e. self-service) [32]; and searches conducted at two different sites [33]. One study also looked at patron engagement with resources identified in literature search results, but it specifically focused on document delivery requests, not immediate online access [34].

### Analyzing literature search request/completion data

Many libraries collect literature search request/completion data such as number of requests, time spent searching, intended use, request topics, and patron categories (e.g. patron affiliation and/or occupation). Libraries can use this data to assist in the design and organization of services [9,10,12], demonstrate value to stakeholders [10,12], determine resource needs [11,12,13], and develop targeted strategies for advancing institutional and library goals for specific groups [9,12].

Like these libraries, the WRHAVL collects data concerning literature search requests. An online LibWizard (Springshare) [35] request form asks patrons the types of resources they are looking for (e.g. journal articles, guidelines, books, or clinical trials), the purpose of their search (e.g. updating guidelines, patient education, writing for publication, professional development), their job title, their location (e.g. hospital, community health office, personal care home), the range of publication dates to search (e.g. last 5 years), and the number of results they would like. When a librarian completes a search, they record additional details concerning the search in LibInsight (Springshare) [36] including the amount of time spent on the search, the dates the search was requested, due, and completed, the resources searched, the topic of the search, and the number of results sent (i.e. the total number of references included in the results list). Although this data can be useful for the purposes listed above, Farrell and Mason claim the utility of these types of usage statistics is “disputed” [20]. This data can measure the efficiency or timeliness of services [37] but cannot fully describe effectiveness or service satisfaction, or explain overall literature search service trends [9,37]. Friesen et al. have suggested that surveys may provide these answers [9].

### Surveys of literature search service users

Surveys “can be a useful way to gather information on client satisfaction with, and use of, a literature search service” [23] and many studies have demonstrated how these services impact patient care and save busy professionals’ time [1]. Surveys have also examined patron satisfaction and preferences concerning a number of different literature search aspects, which has helped libraries to improve their search services [23,25].

Despite these findings, there are concerns with using surveys to evaluate literature search services. One of the issues is participant recall bias [25]. Most studies have tried to mitigate this by using the “‘critical incident technique’ asking respondents to think back on an incident where they used information the library sent them” [20] or by “use of a brief questionnaire sent at the same time as the response” [20]. However, voluntary surveys are also susceptible to non-response bias, where individuals are much more likely to respond if they felt highly satisfied [38]. In addition, the typically low response rate of web-based surveys [28] has led some to question whether there is always sufficient power to detect statistical significance [39].

At the WRHAVL, when reference lists are sent to patrons, the message they receive prompts them to complete a voluntary survey concerning the quality and timeliness of the search, whether they were able to access the resources identified, and whether the instructions to access the resources were clear. Although some studies have suggested sending a questionnaire with the results “is more likely to gain a response” [28], the response rate to these surveys is quite low. For example, for the period examined in this study (October 2022-October 2024), patrons completed a survey for only 7% (n=34/480) of searches sent. In addition to the small sample size, there is some evidence of non-response bias, as individuals who do respond to the library’s survey are often highly satisfied. Ultimately, despite the amount of information collected by the library, the WRHAVL still did not have a good sense of how patrons were engaging with the vast majority of searches completed. To solve this issue, the library developed the novel idea of using a link management tool to generate trackable links for references in literature search results.

After exploring multiple link management options, such as Bitly, the library selected the link management tool Short.io [40] to generate the trackable links. The library updated its VBA macro for literature search reference lists so it would generate trackable Short.io links for each item in the list. These links are included in the literature search results sent to patrons and generate data on which links are clicked. Data from 19 October 2022 to 31 October 2024 was analyzed and provides valuable insights into patron engagement with literature search results over this period.

## Methods

### Literature search requests and data collection

Over the study period 74% (n=354/480) of requests were submitted using the LibWizard request form on the library’s website. Requests that were not submitted using the form were either sent directly to a librarian or the library’s generic inbox. When submitting the form, patrons are required to accept the library’s Conditions of Resource Use, which indicates that their personal information is being collected for the purpose of providing library services and for evaluation. These conditions are also accessible through the Policies section of the library’s website and apply to all library patrons.

Although the library regularly collects and analyzes literature search data for internal service evaluation, the authors consulted with the University of Manitoba’s Research Ethics Board and Manitoba’s Committee for Harmonized Health Impact, Privacy, and Ethics Review to confirm whether ethics review and approval was required for the external publication of this analysis. The ethics review boards determined that ethics review and approval was not required for this study.

### Literature search process

**Fig. 1:**
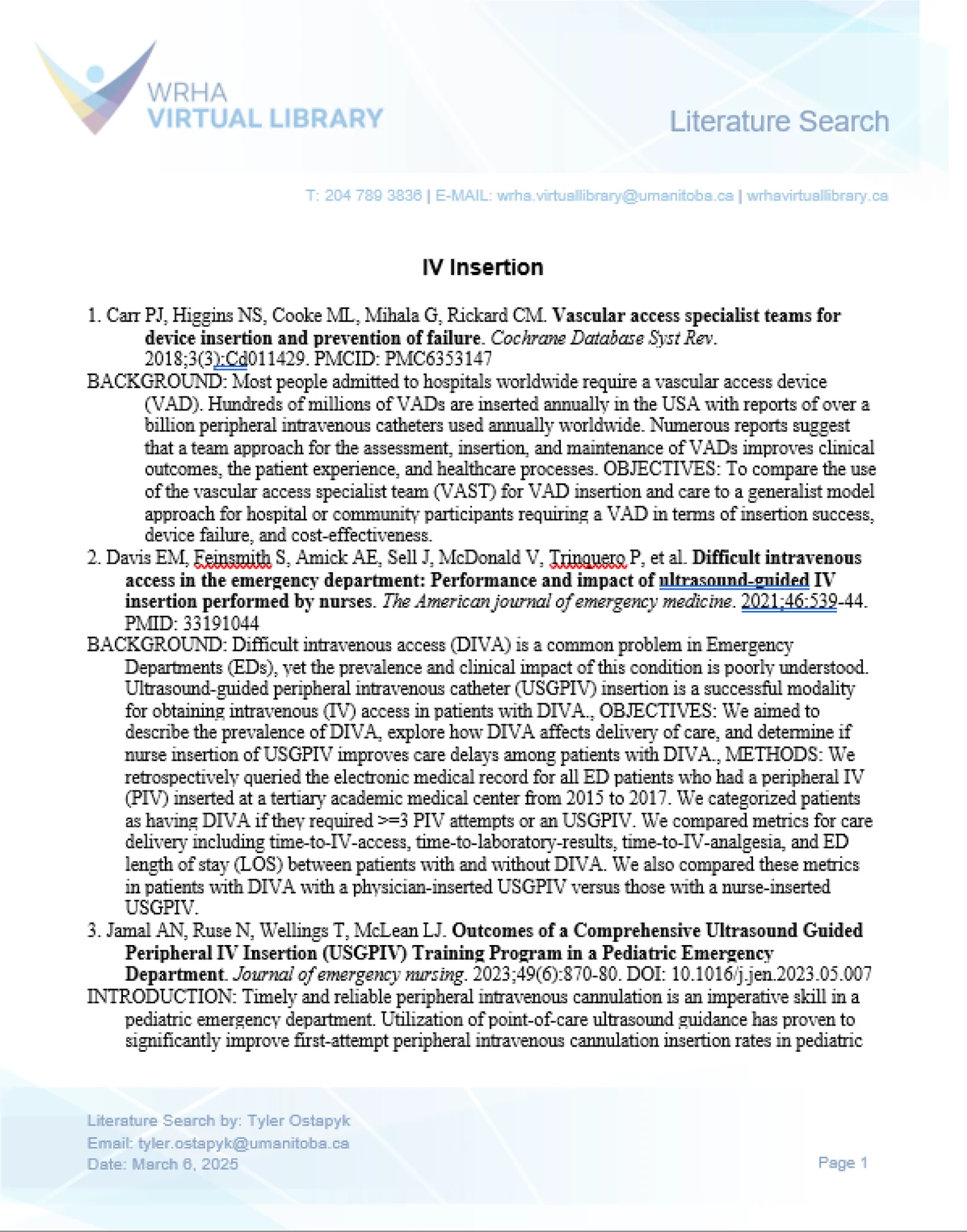
List of references from a completed literature search after the results have been exported from reference management software into Word using the library’s custom template. Each reference includes an abstract and a DOI, PMID, or link to the resource, when available.

When patrons submit a request through the library’s request form, the request is sent to the library’s generic inbox and library staff assign the literature search to a librarian. This librarian conducts the search and saves citations related to the search topic to reference management software (e.g. Zotero (Corporation for Digital Scholarship) [41] or EndNote (Clarivate) [42]). The librarian then exports the references into a Microsoft Word template using a custom output style, which includes DOIs and PMIDs as well as abstracts when available (Figure 1). The librarian saves the Word file with the format [Requestor’s Surname] ~ [Search Topic], for example Smith ~ IV Insertion^*^.

### Generating the Short.io links

After the reference list has been saved, the librarian runs the VBA macro (available at https://osf.io/zv8d9 /files/2yxve) to generate the Short.io links. The macro takes the following steps:

1. Removes all existing hyperlinks from the document.
2. Adds full-text URLs:
  - Searches for DOIs and PMIDs in either labelled format (e.g. DOI: 10.1016/j.ajem .2020.11.013, PMID: 37591703) or URL format (e.g. https://dx.doi.org/10.1016/j.ajem.2020.11.013 or https://pubmed.ncbi.nlm.nih.gov/37591703).
  - Replaces the DOIs/PMIDs found with a custom library-specific LibKey URL based on the DOI/PMID (e.g. https://libkey.io/libraries/2574/gotofulltext/openurl?rft_id=info:doi/10.1016/j.ajem.2020.11.013).
  - Searches for labelled PubMed Central identifiers (PMCIDs) (e.g. PMCID: PMC6353147) and replaces them with a PubMed Central URL based on the PMCID.
  - Searches for UpToDate URLs and adds the library’s EZproxy prefix.
3. Parses the document for all URLs and creates hyperlinks for each URL found.
4. Obtains the file’s current user (i.e. the librarian who created and saved the reference list) to support data validation and troubleshooting, and the search topic (everything that appears after the tilde (~) in the filename).
5. Iterates through each hyperlink, sending the link to the Short.io API along with accompanying metadata (retrieved in Step 4).
6. Retrieves each Short.io link generated through the API and replaces each existing hyperlink with its corresponding Short.io link. The Short.io link generated by the API contains the library’s custom domain (https://go. wrhavirtuallibrary.ca/) followed by a randomly generated six-character string.
7. Changes the “text to display” for DOI, PMID, and PMCID hyperlinks to a labelled format (e.g. DOI: 10.1016/j.ajem.2020.11.013, PMID: 37591703, PMCID: PMC6353147).

### Sending results to patrons and recording the completed search

**Fig. 2:**
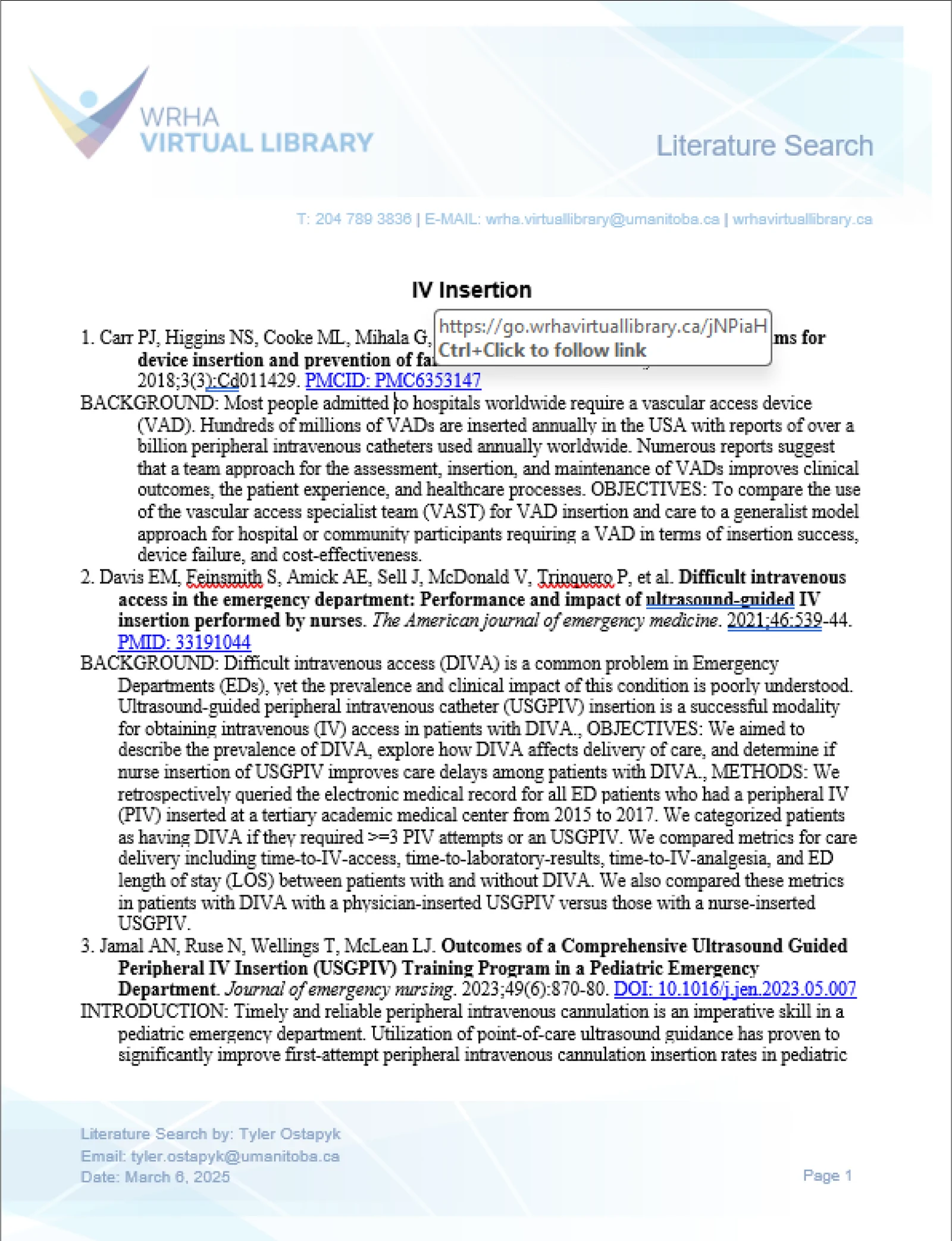
Literature search results after trackable links are generated in Word using the library’s custom VBA macro. PMID, PMCID, and DOI links are displayed in a labelled format with the corresponding ID and the hyperlinks contain the library’s custom Short.io domain followed by a string that is randomly generated by Short.io for each specific link.

**Fig. 3:**
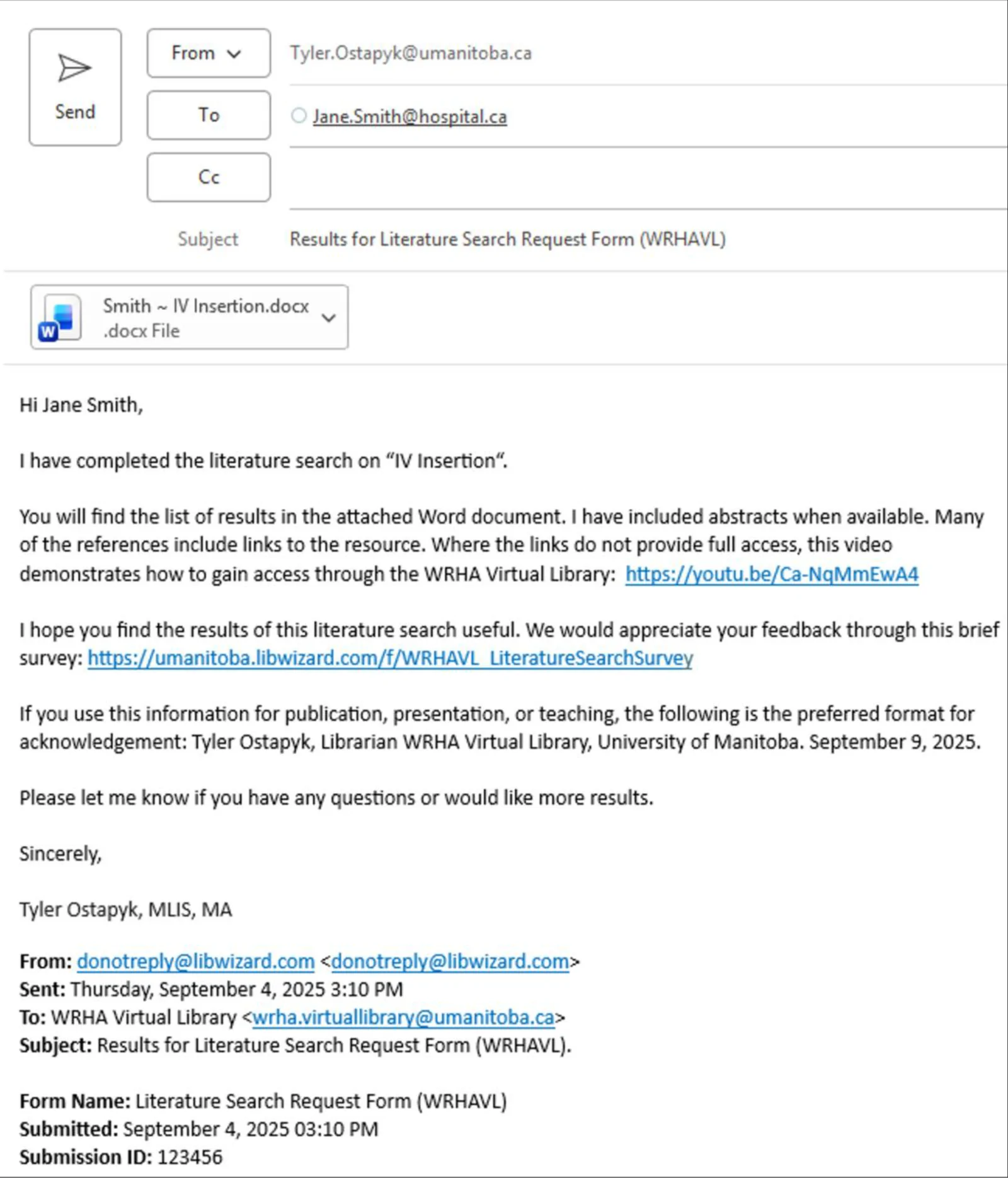
E-mail sent to requestor by the library. The completed reference list in Word format is attached to the e-mail. The message contains instructions on accessing the full-text items using the links and includes an invitation to complete the library’s literature search survey.

After the links are generated, the librarian attaches the file (Figure 2) to an e-mail sent to the requestor. The e-mail (Figure 3) contains the original request form, instructions for accessing full-text, and a prompt to complete the library’s survey. After sending the e-mail, the librarian who conducted the search records the completed search in LibInsight.

### Obtaining and cleaning the data

**Fig. 4:**
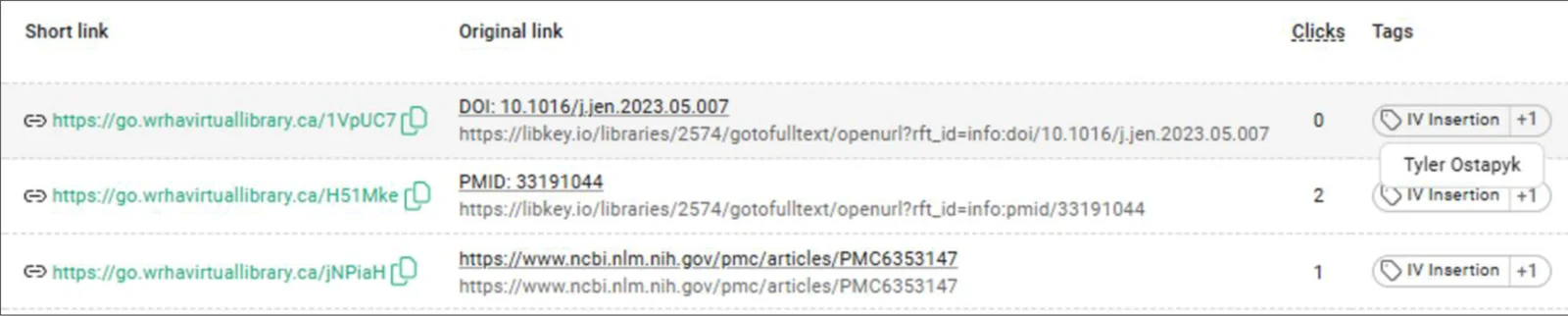
Link data that is automatically collected in the Short.io interface including the original identifier and/or URL, the Short.io generated URL, total number of clicks, and metadata tags.

Whenever a patron clicks a link in the reference list they receive, that click is automatically recorded in Short.io’s interface (Figure 4). The metadata (searcher and search topic) that is passed to Short.io’s API is shown in a Tags column. The interface also offers high-level statistics for the library’s custom Short.io domain (https://go.wrhavirtuallibrary.ca/) such as total number of clicks by date, average number of clicks per link, links clicked the most, top cities/countries where the clicks originated, and top referrers (webpage where the request/click originated, if applicable), as well as individual statistics for each link such as total number of clicks by date, country/city where the click originated, and referrers (if applicable) for each click.

**Fig. 5:**
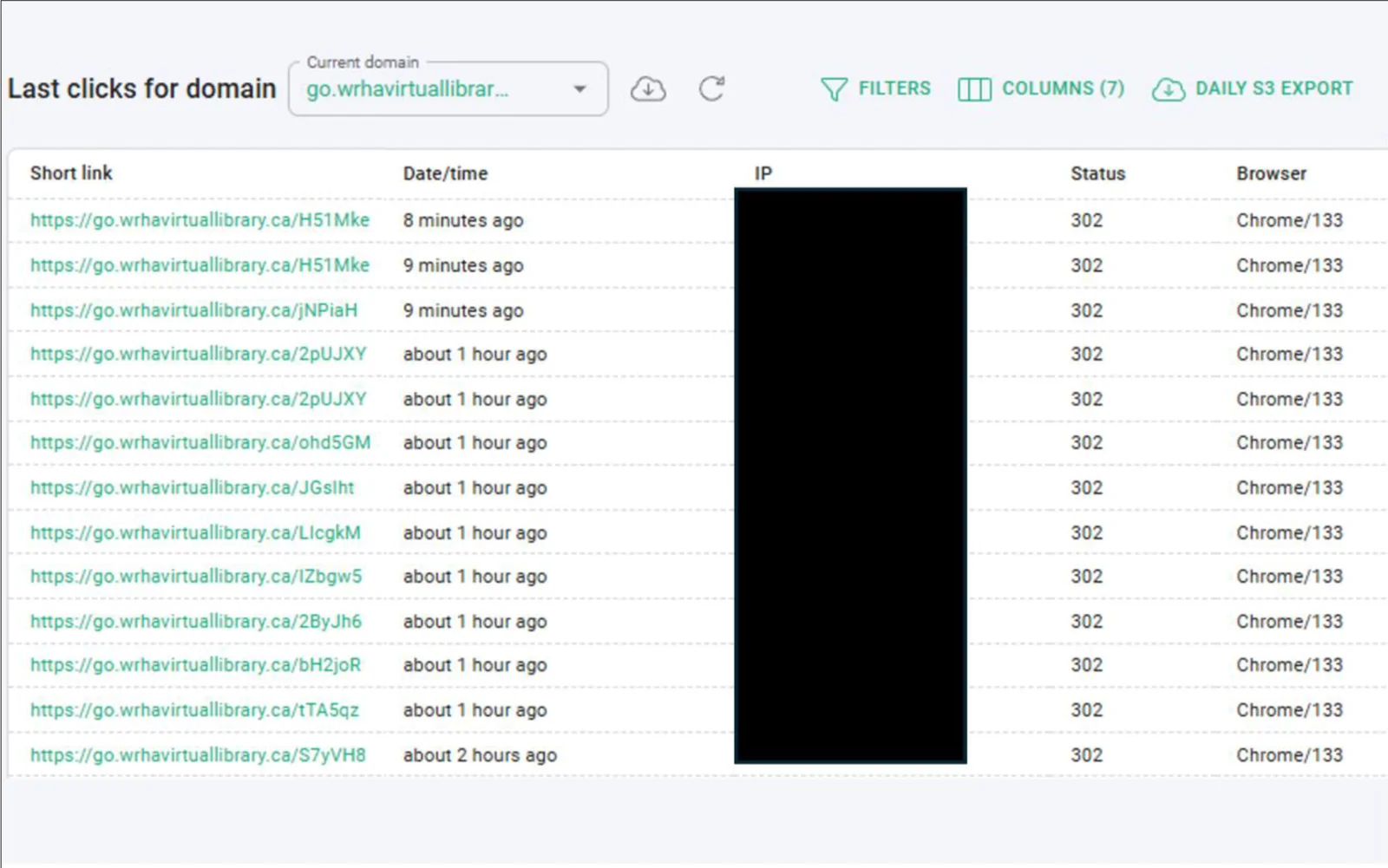
Click data in the Short.io interface. This data includes the IP address where the click originated, the Browser used, and the Short.io URL. IP addresses have been blocked in the figure.

There is a separate section of the Short.io interface that provides data at the click level, indicating when each link was clicked, as well as the IP address of the clicker (Figure 5). IP addresses are collected by Short.io for fraud prevention and troubleshooting [43].

When first examining the click data, the authors discovered clicks from countries that were unlikely to be associated with the library’s patrons. After further investigation, and discussion with representatives at Short.io [44], the authors determined that crawlers and email clients (e.g. Outlook) that scan links in incoming e-mails were likely responsible for these “false clicks.” To try to ensure that only valid clicks by library patrons were included in the data, the authors evaluated the IP addresses using the NordVPN IP Lookup tool [45]. Short.io has a built-in function that allows the user to exclude particular IP addresses from statistics. When a suspected crawler or invalid source was found, that IP address was excluded from the data. Although the WRHAVL is situated at the health campus at the University of Manitoba, nearly all of the library’s patrons are located off-campus at hospitals and other facilities in Winnipeg. Given this, on-site and VPN campus IP addresses were excluded so librarians could test the links before the results were sent without creating “false clicks.”

**Fig. 6:**
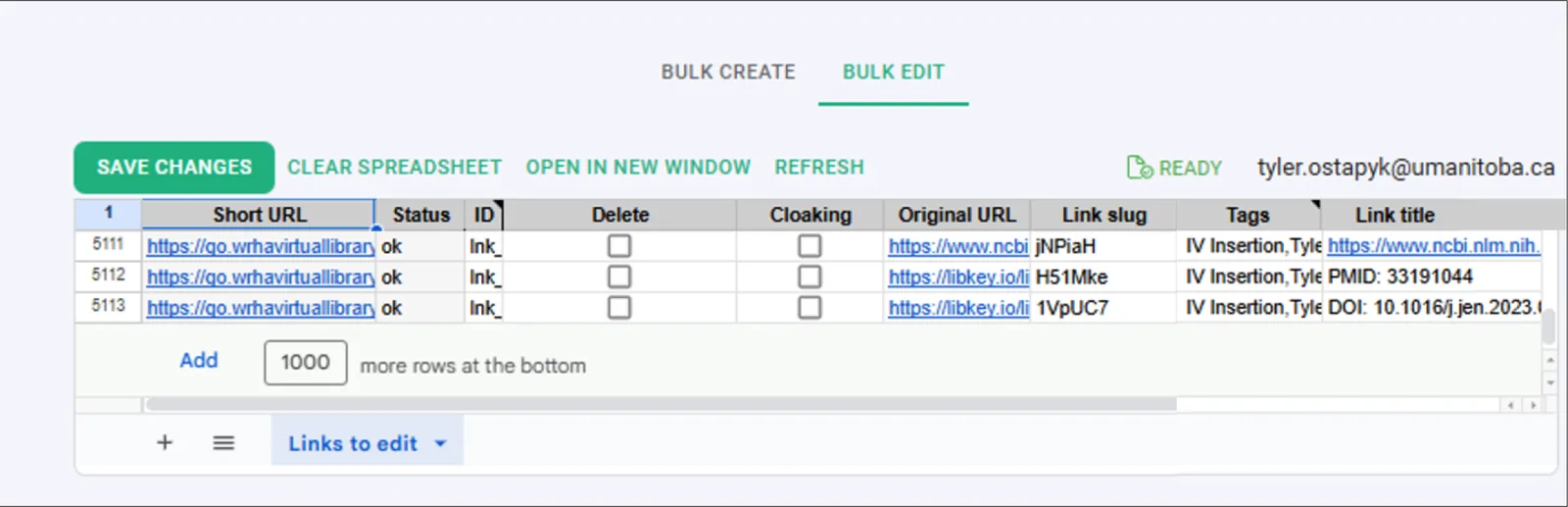
Link data in Google Sheets accessed through the Short.io interface. This data includes the original URL, Short.io URL, and metadata tags, but does not include total number of clicks.

Once cleaned, both the link and click data were copied into Excel for analysis. Short.io has Google Sheets integration for the link data (Figure 6). The authors used this feature to export the data concerning each link for analysis. The click data was exported directly from the interface in CSV format.

### Data analysis

The link data available in the Google spreadsheet does not contain the click numbers for each link, and the click data does not contain the metadata tags generated by the macro. For this reason, both sets of data were cross-referenced in Excel using the unique Short.io URLs. This made analysis of the data at the individual link and literature search level possible.

Librarians sometimes provide multiple unique identifiers and links for the same reference (e.g. PMIDs and DOIs). To account for this, the authors examined the library’s LibInsight data to determine the total number of references included in each search. In this way, the number of clicks could be compared to both the total number of references and the total number of links included in the search. Literature search topics in the LibInsight data were also matched to the corresponding search topic metadata in Short.io to identify searches for which no Short.io links were generated. Reference counts from searches that did not have a match in Short.io were removed from the total reference count for analysis. There was one search that was completed well before the study period but for which links were generated during the study period. This search and its corresponding click and link data were removed prior to analysis.

To determine the popularity of specific resources, counts were generated based on the original URLs of the resources, which are recorded in the Short.io data. For example, if an original URL contained “www.uptodate” it was counted as an UpToDate link. URL counts were generated for several point of care and quick reference resources commonly included in the library’s search results as well as URLs pointing to the library’s discovery service, and URLs containing DOIs, PMIDs, and PMCIDs (see Table 1 for a list of resource categories and URLs). All other URLs (e.g. links to hospital procedures, association guidelines, etc.) were considered “other links” for the purpose of analysis. Two subcategories of resources were also analyzed together: point of care and quick reference resources, and scholarly resources, which the authors defined as URLs containing DOIs, PMIDs, or PMCIDs.

**Table 1:** URLs used to define resource categories and subcategories

Resource	Original URLs Containing	Author-Defined Subcategory
Clinical Key (e.g. patient education materials, clinical overviews)	www.clinicalkey	Point of Care and Quick Reference Resource
JBI EBP	JBI+Database	Point of Care and Quick Reference Resource
LWW Health Library (including Nursing Made Incredibly Easy Collection)	Lwwhealthlibrary	Point of Care and Quick Reference Resource
UpToDate	www.uptodate	Point of Care and Quick Reference Resource
DOI	https://libkey.io/libraries/2574/gotofulltext/openurl?rft_id=info:doi ^*^	Scholarly Resource
PMID	https://libkey.io/libraries/2574/gotofulltext/openurl?rft_id=info:pmid ^*^	Scholarly Resource
PMCID	ncbi.nlm.nih.gov/pmc/articles/^*^	Scholarly Resource
Primo	permalink/01UMB_INST	No Subcategory
Other links	Any URLs that did not contain any of the above	No Subcategory

* The macro generates these original URLs for references with DOIs, PMIDs, and PMCIDs if they are in either URL-based or labelled formats (e.g. DOI: 10.1016/j.ajem.2020.11.013 or https://doi.org/10.1016/j.ajem.2020.11.013 or https://dx.doi.org/10.1016/j.ajem.2020.11.013)

A logistic regression analysis was performed in R (version 4.5.2) [46]. The analysis was done using the glm function with the family selected as binomial as the response variable (at least one click) is binary. The 10 variables under consideration were number of links, number of references, Clinical Key links, UpToDate links, Lippincott Williams & Wilkins (LWW) Health Library links, Joanna Briggs Institute Evidence Based Practice Database (JBI EBP) links, Primo links, DOI links, PMID links, and PMCID links. The variables scholarly resources links and other links were not considered as they were linear functions of other variables in the model and therefore non-estimable. These variables were used to determine which, if any, increased or decreased the probability of a literature search receiving at least one click. After checking for significant factors, a drop-in-deviance test was performed to test that the eliminated variables were statistically non-significant.

## Results

According to the library’s LibInsight data, between October 2022 and October 2024 library staff completed 480 literature searches. Of these, 344 literature searches, or 72% of completed searches, used the trackable Short.io links. A total of 11743 Short.io links were created for 9345 references (see Table 2).

**Table 2:** Summary data of links and clicks

Link and Click Categories	2023-2024^*^	2022-2023^*^	Total Over 2 Years
**Literature Searches**			
Literature searches with trackable links sent	171	173	344
Literature searches with 1+ clicks	96	98	194
% of searches with 1+ clicks	56%	57%	56%
**Links**			
Links created	6308	5435	11743
Links with 1+ clicks	776	685	1461
% of links with 1+ clicks	12%	13%	12%
Total references	5089	4256	9345
Duplicate links^†^	1219	1179	2398

* Years indicate links created from 19 October 2022 to 31 October 2023, and 1 November 2023 to 31 October 2024. Clicks generated at any time over the two-year period were counted

† Extra links created for a single reference (e.g. PMID link when reference also had a DOI link)

There was little variation in the percent of literature searches with clicks from year to year. 56% (n=96/171) of literature searches containing Short.io links had at least one click in 2023–2024, and 57% (n=98/173) of literature searches containing Short.io links had at least one click in 2022–2023. Library patrons clicked 12% of links created (n=1461/11743), or around 1 in 8 links, over the study period.

### Popular resources

Based on the original URLs, the library was able to analyze the clicks and number of links created for specific resources (Table 3).

**Table 3:** Links and clicks for specific resources

Library Resources	Links Created	Clicks	% Clicked
Lippincott Williams & Wilkins Health Library	18	9	50%
UpToDate	88	33	38%
Joanna Briggs Institute Evidence Based Practice	161	59	37%
Clinical Key direct links*	10	2	20%
Primo (library discovery service)	69	11	16%
PMCIDs	984	139	14%
DOIs	7598	837	11%
PMIDs	1799	102	6%
Scholarly resources	10381†	1078†	10%‡
Other links§	1016	269	26%

* Count of ClinicalKey direct links by resource type: 6 patient handouts, 2 drug monographs, 1 clinical overview, and 1 book chapter

† Total of DOIs, PMIDs, PMCIDs

‡ Average of DOIs, PMIDs, PMCIDs

§ Total of links created that did not fall into any of the other categories

**Fig. 7:**
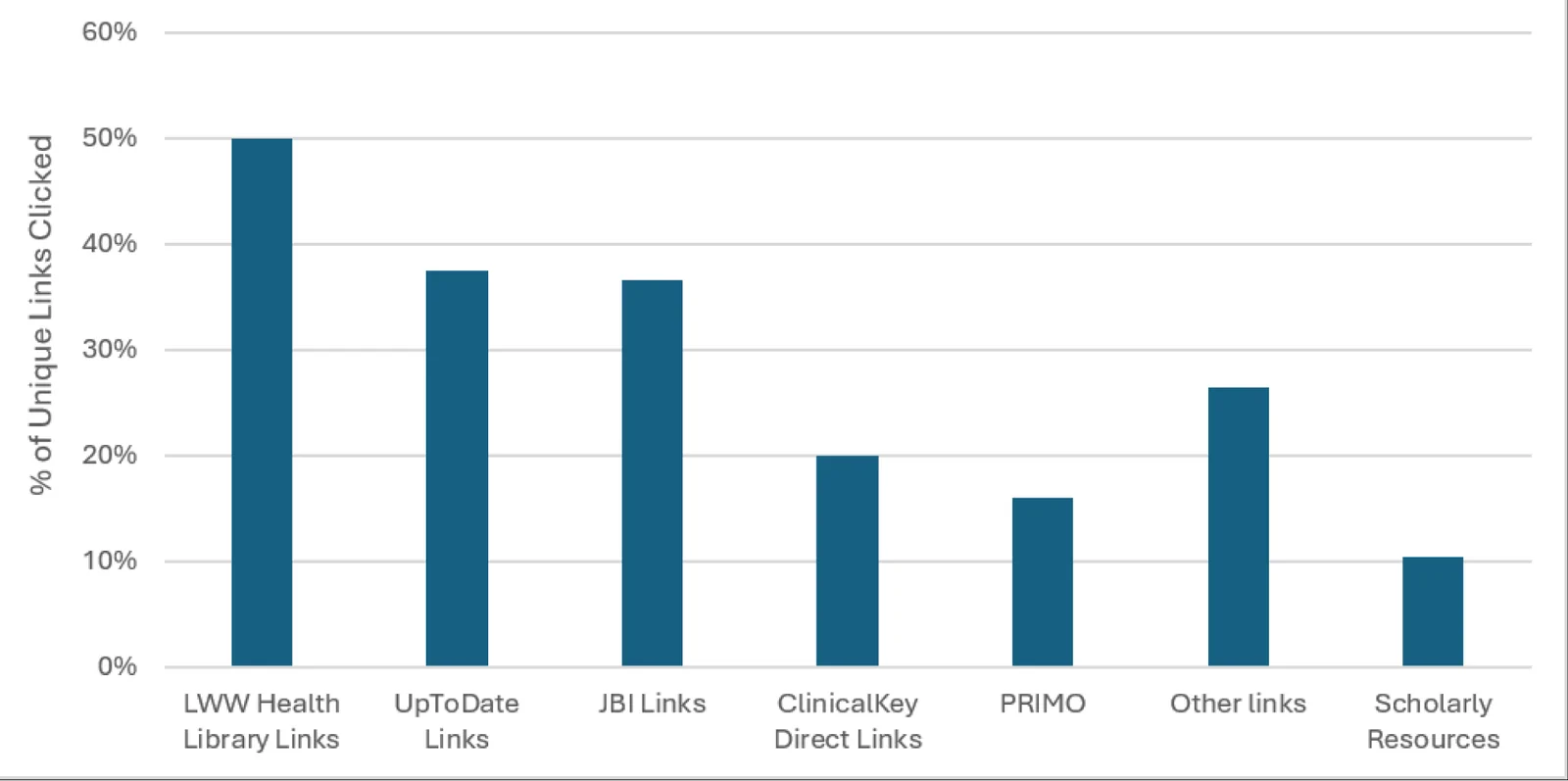
Chart of the percentage of unique links that received at least one click by resource category. The Scholarly Resources category is an average for DOI, PMID, and PMCID generated links so these can be compared as a group with the other categories.

**Fig. 8:**
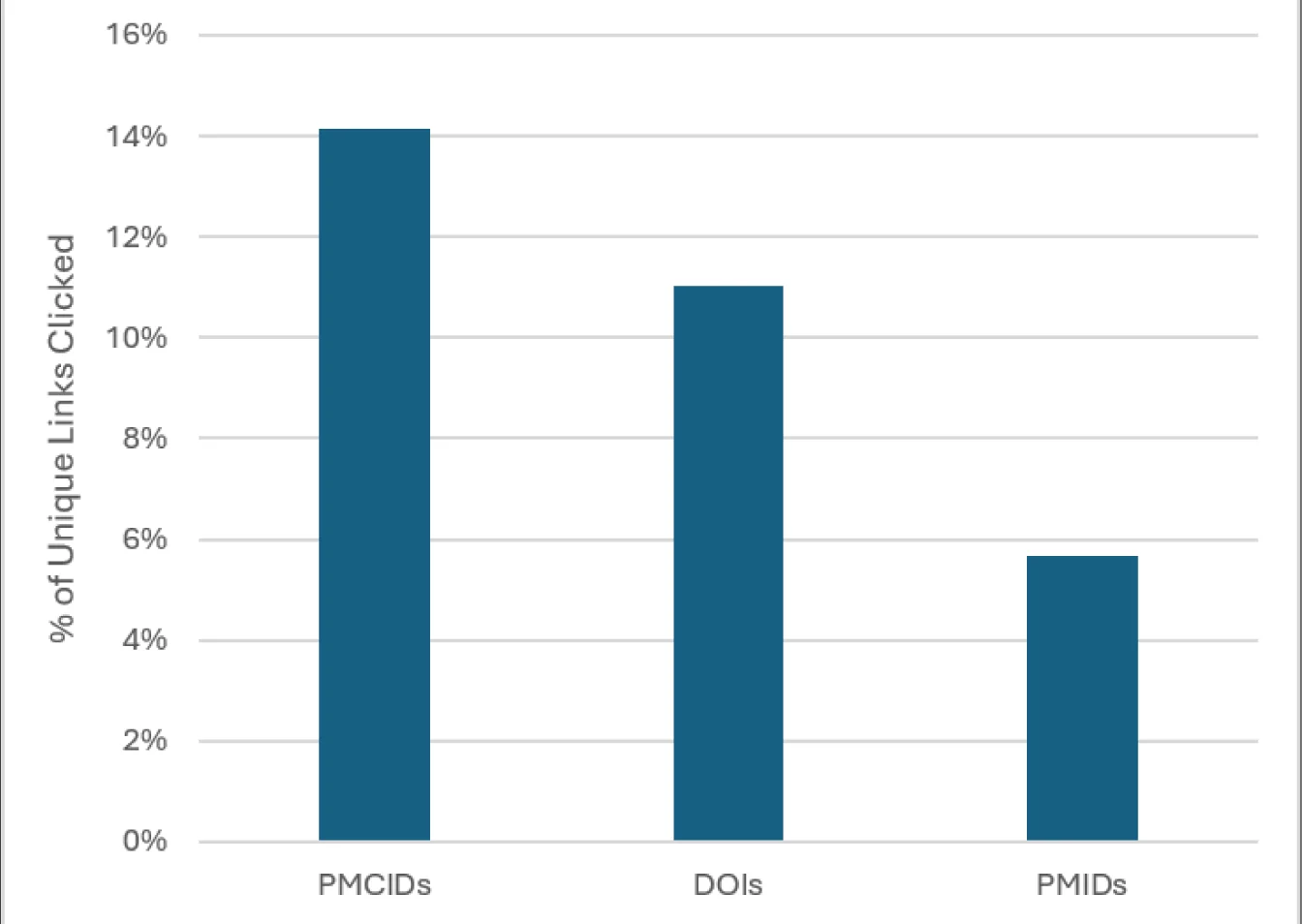
Chart of the percentage of unique links with at least one click in the Scholarly Resources category. These are links that were generated using DOI, PMID, or PMCIDs.

A much higher proportion of links for point of care and quick reference resources had at least one click in comparison to scholarly resources (Figures 7 and 8). Whereas only 10% of scholarly resources links were clicked (n=1078/10381), 50% (n=9/18) of LWW Health Library links, 38% (n=33/88) of UpToDate links, and 37% (n=59/161) of JBI EBP links received at least one click.

### Click trends

**Fig. 9:**
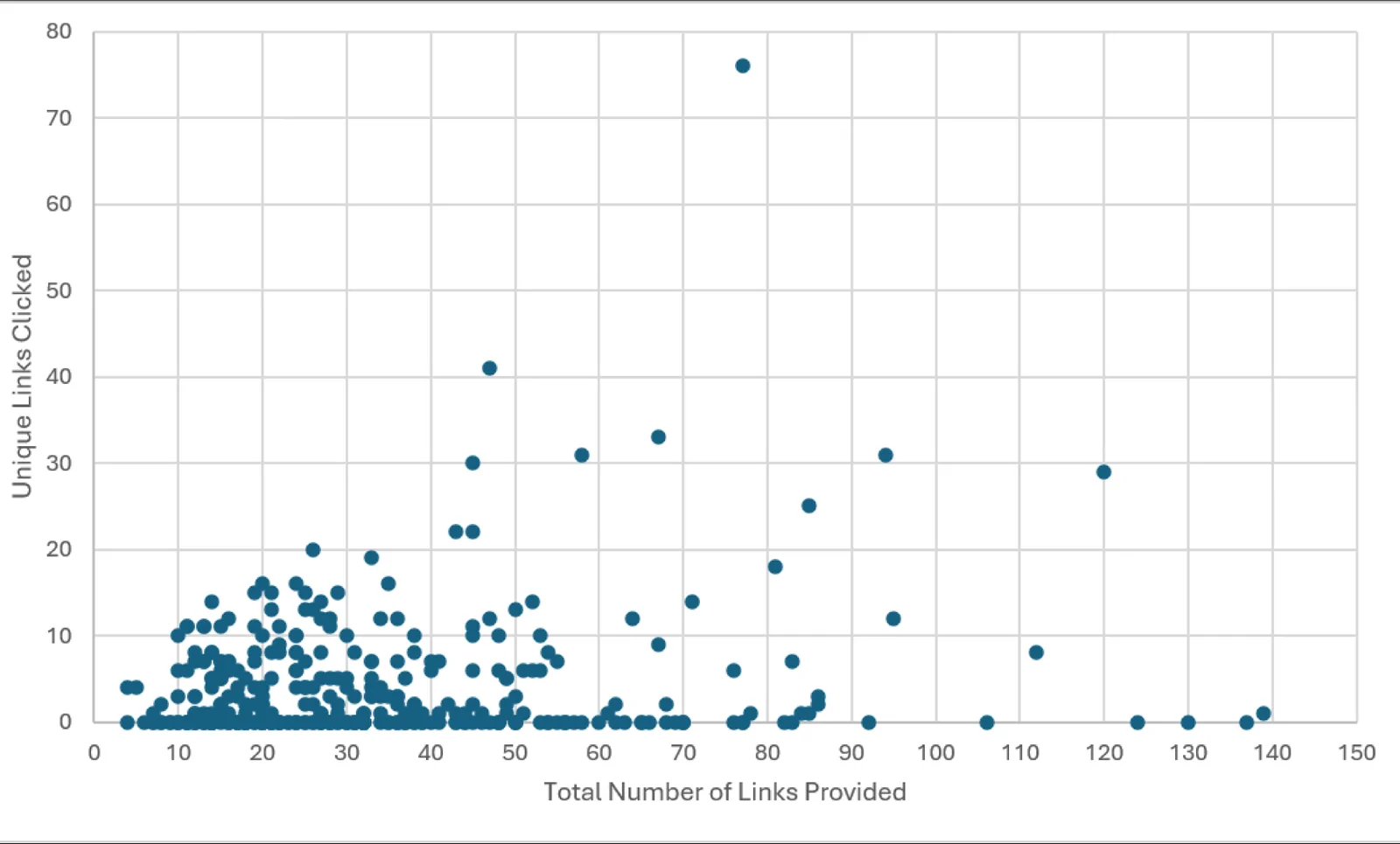
Chart of the total number of unique links that received at least one click by the total number of unique links provided for each literature search.

Analysis of unique links clicked and total number of links provided in each literature search found that only 3% (n=10/344) of recipients clicked more than 20 links in a search, regardless of the total number of results provided (Figure 9).

**Fig. 10:**
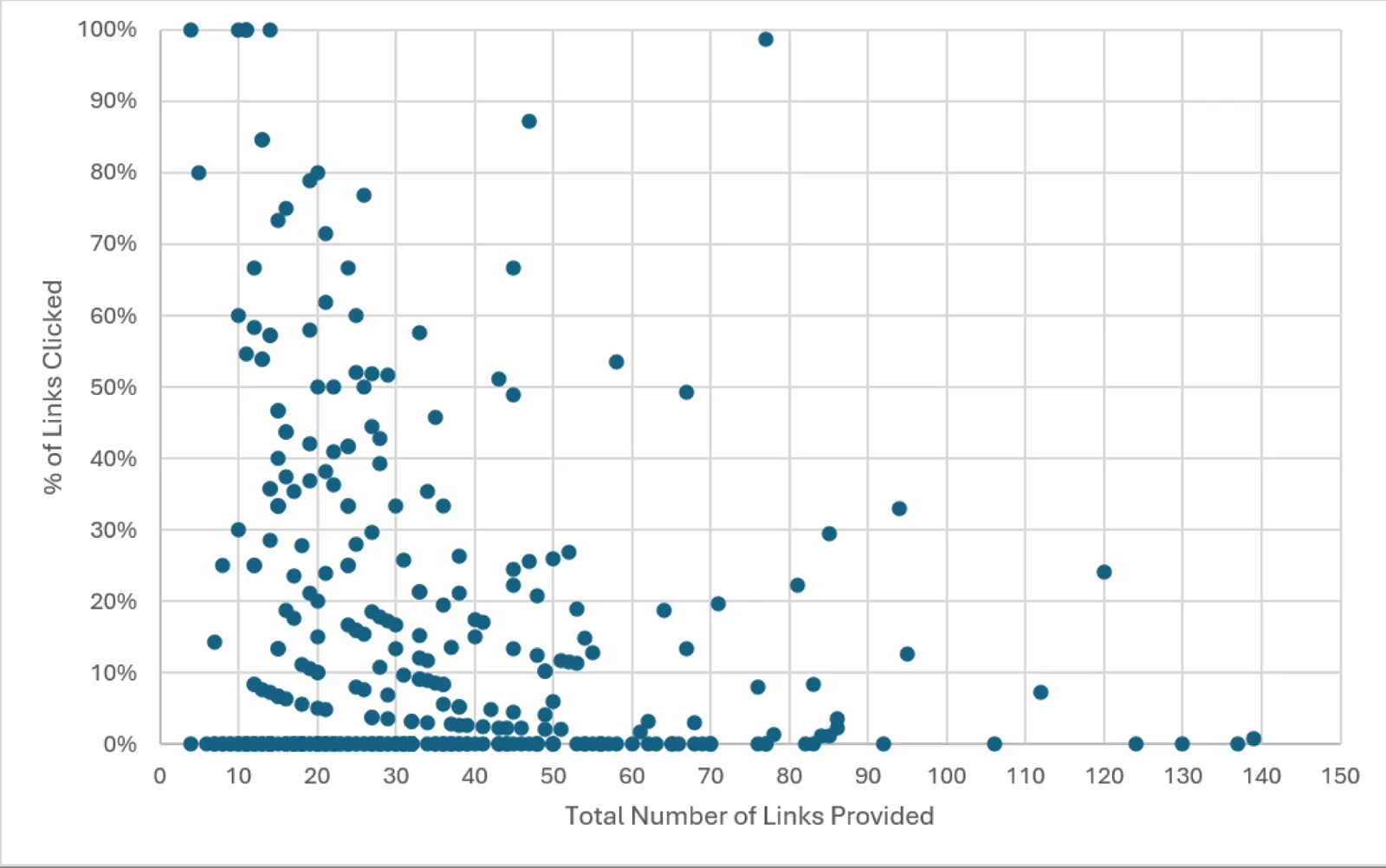
Chart of the percent of unique links that received at least one click by the total number of links provided for each literature search.

Individuals also clicked a higher proportion of links more frequently when a lower number of total links were given. For example, for literature searches that had at least one click, searches that had 1–30 links had at least half of their links clicked in 30% (n=32/108) of cases, whereas only 7% (n=6/86) had at least half of their links clicked if there were more than 30 links in the list (Figure 10).

### Determinants of literature search engagement

A logistic regression analysis was performed with the glm function and binomial family argument for the link function to test for significant variables in a model for a literature search receiving at least one click. Table 4 lists the variables tested, their regression coefficient (the log of the odds ratio), the standard error of the coefficient, a 95% confidence interval for the coefficient, and the p-value for the Wald test to test if they were non-zero and statistically significant.

**Table 4:** Results of logistic regression analysis to test for significant variables that led to at least one click in a literature search

Variable	β_i_ = log(OR)	Std. Error	95% CI		P-value
(Intercept)	0.194060	0.211596	-0.2206682	0.6087882	0.3591
Number of Links	0.107343	0.056861	-0.00410146	0.2187906	0.0591*
Number of References	-0.051382	0.041496	-0.1336236	0.0308596	0.2156
Clinical Key Links	-0.745710	0.496695	-1.7192322	0.2278122	0.1333
UpToDate Links	0.236151	0.235478	-0.2253859	0.6976879	0.3159
LWW Health Library Links	0.296157	0.507385	-0.6983176	1.2906316	0.5594
JBI EBP Links	-0.006228	0.109811	-0.2214576	0.2090016	0.9548
Primo Links	0.032022	0.131342	-0.2254083	0.2894523	0.8074
DOI Links	-0.061589	0.033552	-0.1273509	0.0041729	0.0664*
PMID Links	-0.120066	0.0553585	-0.2286206	-0.0115114	0.0302*
PMCID Links	-0.085247	0.062793	-0.2083213	0.0378273	0.1746

* Indicates a p-value <0.10

As there were many variables in the full model and likely issues with multicollinearity, due to only one p-value being less than 0.05, the authors first selected all variables that had p-values less than 0.10 (Number of Links (0.0591), DOI Links (0.0664), PMID Links (0.0302)) to propose a reduced model that only contained those three variables. The logistic regression was then rerun on this model with the output summarized in Table 5.

**Table 5:** Results of reduced model logistic regression analysis run on variables that had p-values less than 0.10

Variable	β_i_ = log(OR)	Std. Error	95% CI		P-value
(Intercept)	0.20516	0.20221	-0.1911716	0.6014916	0.31031
Number of Links	0.04698	0.01858	0.0105632	0.0833968	0.01145
DOI Links	-0.05175	0.02197	-0.0948112	-0.0086888	0.01852
PMID Links	-0.07650	0.02640	-0.1282440	-0.0247560	0.00376

In this reduced model, all regression coefficients had statistically significant p-values (p<0.05) for the Wald test. To test that the other variables should have been removed, a drop-in-deviance test was performed and had test statistic G = 461.86 – 454.94 = 6.92. The test statistic follows a chi-squared distribution with 7 degrees of freedom and p-value 0.4372568. As the p-value is greater than 0.05, there is insufficient evidence the coefficients for the removed variables were nonzero given number of links, DOI links, and PMID links were in the model suggesting they could be removed. With all variables in the reduced model testing as statistically significant at the 0.05 level, justifying the initial threshold of 0.10, the final proposed statistical model is:


$$log(\hat{odds})\;=\;0.20516\;+\;0.04698\left(Number\;of\;links\right)-0.05175\left(DOI\;links\right)-0.07650\left(PMID\;links\right)$$

This analysis found that when the other variables were held constant, total number of links predicted an increase in the odds of someone clicking at least one link in a reference list, and the number of both DOI and PMID links predicted a decrease. The finding concerning DOI and PMID links suggests that when a larger percentage of total links given are not DOI or PMID links, there are increased odds that someone will engage with the literature search results.

For example, if the following three reference lists are provided, the logistic regression analysis found that there are increased odds that a patron will click at least one link in list #2:

1. 20 links – 8 DOI links, 8 PMID links, 4 other links
2. 20 links – 2 DOI links, 2 PMID links, 16 other links
3. 20 links – 15 DOI links, 5 PMID links

## Discussion

### Notable findings

#### Encouraging patron engagement with literature search results

The results suggest that the library’s patrons more frequently click on links to quick reference or point of care resources and that there are increased odds they will access the library’s resources if the reference lists contain a higher percentage of links that are not DOI or PMID links. This suggests that librarians can encourage patron engagement with literature search results by ensuring they include a good number of non-scholarly references in their search results when relevant to the requestor’s interest. The library could consider adding “point of care” as an option to the types of resources section of the request form to better assess when including these resources is most appropriate. It would also be interesting to determine whether this pattern differs across other request form categories such as purpose of search, job title, and status (i.e. affiliation). This may be explored in a future study.

#### Ideal number of total references

The results suggest that patrons tend to not click more than 20 links in a reference list, although no ideal number of total references was determined. Analysis of the 2022–2023 data [47] seemed to suggest that the total number of clicks a reference list received began to decrease when more than 30 references were provided in a list, and that total clicks were highest for lists with 20–30 references. However, this trend was less prominent in the 2023–2024 data and was not determined to be significant after further analysis.

### Limitations

#### Influence of abstracts

Some of the references included in the lists do not have abstracts and it is possible that links for these resources are clicked more frequently because information about them is not readily available in the reference list. As point of care and quick reference resources generally do not have abstracts, this could potentially account for the higher proportion of clicks for these resources. Although it is theoretically possible to pass metadata concerning abstracts to the Short.io API, the library has not yet found a way to differentiate references with abstracts from those without abstracts using the macro. This may be worth exploring in the future.

#### Multiple links for the same reference

When librarians compile reference lists that contain PMID and DOI links for a single reference, the DOI link appears at the end of the line, which may prompt patrons to click this link more often. This may account for the lower proportion of clicks for PMID links. Developing the macro so it is able to flag cases where there are two links for the same reference would enable the library to further investigate this, but the library has not done so at this point.

#### Resource categorization

Resource categories were selective author-defined categories based on original URLs. It is possible that some resources that could be considered scholarly resources did not have a DOI, PMID, or PMCID URL and were instead categorized as Primo or “other links.”

#### Literature searches with no clicks

Although patrons clicked at least one link in 56% (n=194/344) of literature searches sent with trackable links, this means patrons did not try to access a single resource through the links provided in 44% (n=150/344) of searches. Possible explanations include that patrons prefer to access items through some other method (e.g. through Primo, document delivery, or PubMed), or that some patrons only read abstracts in reference lists rather than access the full text. Based on library account information, approximately 10% of the library’s patrons also have student, study program, or faculty cross-appointments at the University of Manitoba and may have clicked links provided in reference lists while on campus. As campus IP addresses were blocked to remove librarians’ test clicks, it is possible that some legitimate on-campus clicks were excluded from the data. The library could explore alternative approaches to testing links to enable tracking of on-campus links in the future.

It is also possible that some literature searches received clicks after the data was pulled for analysis, especially if the search was completed near the end of the study period. Currently, the literature search links do not expire, but any links clicked after 31 October 2024, were not included in the analysis. As links could be clicked at any time in the future, even years after the original literature search was sent, it was necessary to use a specific cut-off date for the analysis.

#### Searches without trackable links

Only 72% (n=344/480) of searches completed over this period were sent with trackable links and included in the analysis. As the macro is designed for use in Word, links could not be easily generated when patrons requested results in another format, such as RIS. Additionally, in rare cases the macro encountered an issue with the reference list, or the Short.io API was down or not working as anticipated, and literature searches were sent without the shortened links.

#### Privacy concerns

Although Short.io is General Data Protection Regulation [48] compliant and does not share the data they collect with third-party services [49], there could be potential privacy concerns surrounding the collection, storage, and analysis of patron IP addresses. Although most of the IP addresses Short.io collects are from the patrons’ institutions (e.g. hospitals), if the patron clicks a link on a personal network outside of their workplace the general location where the click originated may be potentially identifiable. To protect patron privacy, the library did not manipulate or analyze patron IP addresses in any way beyond using the option in the Short.io interface to exclude certain IP addresses from the data. IP addresses were not used to identify individuals or groups for the purposes of this study and the IP address data will not be shared outside of the library. Click data, including IP addresses, is automatically deleted from Short.io’s server after one year [50].

#### Technical concerns

Since beginning this process there have occasionally been changes to the Short.io API that were not immediately identified and have required revisions to the macro. Any library using a similar method should be aware of this and monitor for changes to the API.

### Conclusion

In addition to improving patron access to full-text links, the library’s link generation process has provided useful information concerning patron engagement with the WRHAVL’s literature search service. The library will continue utilizing this process and intends to analyze the data on a yearly basis to glean new insights into patron engagement with this service. Opportunities for further investigation include determining why some patrons do not click a single link, looking at whether inclusion or exclusion of abstracts affects click frequency, and further examining the relationship between total number of references provided and number of clicks. Other libraries are welcome to adapt the process and macro for their own literature search services.

## Data Availability

Summarized click and link data for each literature search can be found on Open Science Framework at https://osf.io/zv8d9/files/6wr2x. The VBA macro used to generate the trackable links is available under a GNU general public license and can be found at https://osf.io/zv8d9/files/2yxve.

## Footnotes

^*^The IV insertion literature search used as an example throughout this paper was created by the authors for the purposes of demonstrating the literature search process and was not an actual literature search completed by the library.
